# Protease-serpin-microbiome interactions in inflammatory bowel disease: toward integrated biomarkers of gut health

**DOI:** 10.1080/19490976.2026.2726641

**Published:** 2026-09-02

**Authors:** Meshal Almalki, Camilla R. De Pierri, Tristan Méric, Héla Mkaouar, Donia Slimani, Vincent Mariaule, Sirine Tebassi, Nizar Akermi, Emmanuelle Maguin, Juan Hernandez, Roberto T. Raittz, Moez Rhimi, Francesco Asnicar

**Affiliations:** a Université Paris-Saclay, INRAE, AgroParisTech, Micalis Institute, MIHA Team, Jouy-en-Josas, France; b Department of Cellular, Computational and Integrative Biology, University of Trento, Trento, Italy; c Oniris VetAgroBio Nantes, Nantes, France; d Laboratory of Artificial Intelligence Applied to Bioinformatics, Universidade Federal do Paraná, Curitiba, Brazil

**Keywords:** Inflammatory bowel diseases, serine proteases, serpins, human gut microbiome, host-microbiome axis

## Abstract

Proteolytic imbalance involving host and microbial serine proteases and their inhibitors (serpins) contributes to intestinal barrier disruption and chronic inflammation in inflammatory bowel disease (IBD). However, the interplay among these components remains insufficiently characterized. Here, we survey 13,304 publications over five decades, to map protease-serpin-microbiome literature in IBD. Our analysis reveals a fragmented literature structure, with uneven coverage and limited cross-domain integration among host proteases, microbial proteases, and inhibitory pathways. We synthesize evidence linking these components to intestinal barrier integrity, mucosal immunity, extracellular-matrix remodeling, and microbial ecology. Considering the protease-serpin-microbiome axis may provide an integrative direction for future studies of IBD pathogenesis, including the investigation of mechanisms underlying disease heterogeneity, prioritizing future biomarker and therapeutic studies.

## Graphical abstract

**Figure:**
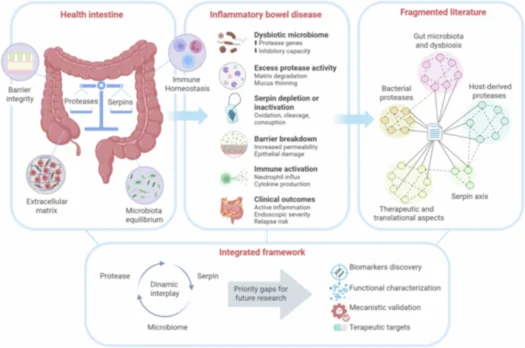

## Introduction

Intestinal homeostasis depends on continuous interactions between the epithelial barrier, the mucosal immune system, and the human gut microbiome, which preserve tissue integrity while allowing the intestine to cope with constant exposure to microorganisms and dietary antigens.^1^ ^,^ ^2^ Proteolysis, mediated by proteases, contributes to tissue remodeling, epithelial renewal, extracellular-matrix turnover, and immune regulation,^3^ however, excessive or uncontrolled protease activity can damage host tissues and promote inflammation.^3-5^ The proteolytic activity is controlled by a set of endogenous inhibitors, like Alpha-1-antitrypsin (AAT), a circulating serpin mainly produced by the liver, which primarily inhibits neutrophil elastase, a neutrophil-derived serine protease involved in inflammatory tissue damage.^6-8^ By contrast, elafin and secretory leukocyte protease inhibitors (SLPI) are mucosal whey acidic protein (WAP) domain protease inhibitors that act at the intestinal epithelial surface and inhibit selected neutrophil- and epithelial-associated serine proteases.^6^ ^,^ ^9-11^ Other antiprotease families, such as tissue inhibitors of metalloproteinases (TIMPs) and cystatins, provide additional regulatory layers.^12^ Together, these inhibitor systems contribute to the regulation of proteolytic activity in the intestinal mucosa.^13^ Disruption of this proteolytic balance has been described in several host health conditions, including irritable bowel syndrome, celiac disease, and inflammatory bowel disease (IBD),^5^ ^,^ ^14^ and comparable proteolytic and serpin dysregulation has also been reported in colorectal cancer.^15^ ^,^ ^16^ Among these, IBD offers a relevant context to investigate this proteolytic imbalance, as it combines chronic mucosal inflammation, epithelial barrier disruption, dysbiosis, tissue remodeling, and increased exposure to luminal proteolytic activity.

IBD is a collective term encompassing chronic, relapsing inflammatory conditions of the gastrointestinal tract.^17^ The two major recognized forms of IBD are ulcerative colitis (UC) and Crohn’s disease (CD), which differ in their anatomical distribution, depth of tissue involvement, and histopathological features.^18^ ^,^ ^19^ UC typically involves continuous mucosal inflammation extending proximally from the rectum, whereas CD is segmental, may affect any gastrointestinal region, and can involve the full thickness of the intestinal wall.^20^ ^,^ ^21^ These anatomical differences extend within each phenotype: extensive/pancolonic versus limited disease in UC, and ileal versus colonic involvement in CD, have been associated with distinct microbial signatures, suggesting that disease location may also shape local proteolytic activity.^22^ ^,^ ^23^ The global incidence and prevalence of IBD have increased markedly over the past few decades, reflecting evolving environmental exposures and westernized lifestyles.^24-26^ Although the etiology of IBD remains incompletely understood, current evidence suggests a multifactorial origin involving a dysregulated immune response in genetically susceptible individuals, triggered by environmental factors and perturbations of the gut microbiome.^27^ ^,^ ^28^

The human gut microbiome, the microbial community colonizing the human gastrointestinal tract, plays a fundamental role in human health,^1^ contributes to digestion, energy harvesting, biosynthesis of bioactive molecules, immune system development, and resistance to pathogen colonization.^2^ ^,^ ^29-32^ Its composition is shaped by lifestyle and diet, among other factors, and is associated with host health and metabolic status.^33^ ^,^ ^34^ In IBD, imbalance of the gut microbiome, with increased abundance of *Pseudomonadota*, particularly *Enterobacteriaceae,* ^35^ alterations in the *Bacillota*/*Bacteroidota* ratio, depletion of short-chain fatty acid (SCFA) producing taxa such as *Faecalibacterium duncaniae* (formerly *F. prausnitzii*) were found as biomarkers.^35-37^ Other studies have reported altered abundances of *Bifidobacterium* and *Enterococcus* species, and *Streptococcus salivarius*, together with depletion of *Eubacterium* species and *Anaerostipes hadrus.* ^38,39^ These taxonomic associations do not by themselves define microbial function: effects can vary with strain-level traits, metabolic activity, disease context, and host environment, so individual taxa should be interpreted within their ecological and functional setting.

Although intestinal inflammation is a common feature of CD and UC,^38-40^ disease-specific patterns have been reported, including reduced *Akkermansia muciniphila* and *Roseburia hominis* in UC,^40^ ^,^ ^41^ and increased abundance of adherent-invasive *E. coli* (AIEC), *Ruminococcus gnavus*, and *Veillonella parvula* in CD.^42-45^ *Fusobacterium nucleatum* has also been associated with both UC and CD, with particularly increased abundance reported in colorectal cancer.^33^ ^,^ ^46^ Beyond taxonomic alterations, multi-omics studies revealed functional dysregulations of the gut microbiome in IBD,^17^ ^,^ ^47-50^ such as altered bile acid metabolism,^51^ ^,^ ^52^ dysregulated bile salt hydrolase activity, affecting mucosal immunity, epithelial integrity, and microbial community structure,^51-56^ and disrupted tryptophan metabolism, impairing immune regulation and increasing susceptibility to intestinal inflammation.^57^ ^,^ ^58^ Conversely, SCFAs which can help reinforce the epithelial barrier function, modulate T-cell responses, and suppress pro-inflammatory cytokines, are reduced in IBD patients.^59^ ^,^ ^60^

The gut microbiome, through microbial protease production, can modify the intestinal proteolytic environment, which could interact with endogenous protease inhibitors, and elevated serine protease activity, coupled with decreased expression of natural inhibitors, has been observed in IBD patients,^61^ contributing to barrier dysfunction^3^ ^,^ ^62^ and promoting inflammation through cytokine and chemokine processing. While host-derived proteases have been extensively studied,^6^ ^,^ ^63^ ^,^ ^64^ microbial proteases remain comparatively less characterized.

Despite major advances in therapy, there is currently no curative therapy for IBD. Existing treatments aim to induce and maintain remission and promote mucosal healing,^5^ ^,^ ^65^ but treatment responses remain heterogeneous.^66^ ^,^ ^67^ Emerging therapies such as Janus kinase (JAK) inhibitors, offer new avenues for refractory disease but still predominantly target immune pathways.^65^ ^,^ ^66^ Although immune-directed therapies may indirectly modify proteolytic activity by reducing inflammation, they were not developed to directly target luminal proteases, microbial protease production, or endogenous protease-inhibitor pathways.

In this context, IBD provides a common ground for investigating epithelial homeostasis, immune regulation, and host-microbiome interactions through the lens of proteolytic regulation. Here, we defined the conceptual landscape of the protease-serpin-microbiome axis in IBD. We combined a text-mining analysis of 13,304 PubMed records with a synthesis of evidence reported in published experimental, clinical, and observational studies, and revealed that host proteases, microbial proteases, and serpins are represented in largely distinct semantic and co-retrieval structures, indicating conceptual fragmentation within the analyzed literature. This fragmentation provided the organizing rationale for the review. We therefore brought together evidence on host proteases, microbial proteases, serpins and other endogenous inhibitors, and microbiome-associated mechanisms that are commonly represented in separate conceptual domains.

## Materials and methods

### Literature search and filtering

We queried PubMed (https://pubmed.ncbi.nlm.nih.gov/) on March 31^st^, 2026, with the following query text: (Inflammatory Bowel Disease[Title/Abstract] OR Inflammatory Bowel Diseases[Title/Abstract] OR Inflammatory Bowel Diseases[MeSH Terms] OR Crohn's Disease[MeSH Terms] OR Crohn disease[Title/Abstract] OR Crohn diseases [Title/Abstract] OR Ulcerative colitis[Title/Abstract] OR Colitis, ulcerative[MeSH Terms]) AND (protease[Title/Abstract] OR proteases[Title/Abstract] OR serine proteases[MeSH Terms] OR Protease Inhibitors[MeSH Terms] OR serpin[Title/Abstract] OR serpins[Title/Abstract] OR serpins[MeSH Terms] OR microbiome[Title/Abstract] OR microbiomes[Title/Abstract] OR microbiota[Title/Abstract] OR microbiotas[Title/Abstract] OR microbiota[MeSH Terms] OR metagenomics[Title/Abstract] OR metagenomic[Title/Abstract]). This yielded a total of 13,877 articles. Bibliographic metadata included PubMed identifier (PMID), title, abstract, full author name, and publication date.

Retrieved records were processed through a custom MATLAB pipeline (see Data Availability) for data filtering, after the concatenation and normalization of the titles and abstracts, we required a minimum text length of 300 characters, and duplicate entries were removed. These filters yielded a final curated corpus of 13,304 publications spanning six decades of literature (1960–2026).

### Text preprocessing and embedding generation

We analyzed the curated corpus using a vector text mining framework implemented in MatLab (R2012b) and adapted from Ieger-Raittz et al.^68^ and Machado et al.^69^

*Pre-processing steps.* Each document was converted to uppercase, tokenized at the word level removing non-alphabetic characters and English stopwords, retaining only letter-based tokens (e.g., the sentence “INFLAMMATORY BOWEL DISEASE AFFECTS THE GUT MICROBIOME.” was pre-processed as: “INFLAMMATORY”, “BOWEL”, “DISEASE”, “AFFECTS”, “GUT”, “MICROBIOME”). For term enrichment, frequencies were computed for each word to identify domain-enriched vocabulary (Supplementary Figure S1a).

*Embedding generation.* The BIOTEXT^69^ framework converts each text into a biological-like format, where text characters are mapped to representations analogous to amino acids. The SWeeP (Spaced Words Projection) model^70^ scans each encoded sequence by applying a spaced-word mask (1-1-0-1-1) as a sliding window across the sequence. Positions marked with “1” are retained to form spaced-word instances, while positions marked with “0” are ignored, enabling non-contiguous pattern representation. The resulting spaced-word occurrences are aggregated into a high-dimensional feature matrix that captures positional and compositional structure. This matrix is subsequently projected into a 1,369-dimensional vector space, referred here as SWeeP representation, yielding a fixed-length representation for each text used in downstream analyzes (Supplementary Figure S1b).

Beyond the numerical representations, the pipeline generates two browsable HTML outputs: *(i)* WORDS.html, which indexes domain-enriched terms (i.e., words or word-based units extracted from the text) and, for each term, provides its embedding-related words, related abstracts, a local term-centric dendrogram linking it to its neighboring terms in embedding space (computed from a pairwise distances among terms represented in the first 50 PCA components using neighbor-joining (NJ) method^71^ and a generalized Minkowski metric), and a year-usage plot; *(ii)* TEXTS.html, which indexes all curated publications with one row per document and links to the full title and abstract files, functioning as the browsable document corpus. Both WORDS.html and TEXTS.html output files are provided as Supplementary Files.

A global corpus-level NJ dendrogram was built from pairwise distances among up to 1,500 selected domain-enriched terms where each term was represented by the first 300-dimensional PCA components of its word embedding derived from the original 1,369-dimensional SWeeP representation. Terms were prioritized using a weighted combination of document frequency and a rank derived from publication year (temporal component).

### Logical expression framework

To map thematic concepts within the curated IBDs corpus at the level of interpretable, retrievable units, we used Boolean queries termed logical expressions (LOGEXPs). A LOGEXP is a Boolean combination of keywords that identifies documents addressing a specific concept, enabling systematic quantification of research focus while accounting for terminology variation.^68^ The LOGEXP analyzes, corpus statistics, and figure generation were implemented in Python (version 3.13.9).

*Concept selection.* We translated the exploratory browsing of the corpus into operational retrieval rules, using the global word dendrogram by clade-level inspection to define thematic macro-groups. Each macro-group was operationalized as a LOGEXP which was iteratively refined by incorporating additional mechanism and disease-relevant terminology and synonyms identified through a targeted, manual literature review,^72^ aiming to improve retrieval without compromising semantic specificity. The resulting LOGEXP set was organized into thematic dimensions aligned with the macro-thematic groups. The final set comprised 49 LOGEXP queries, reflecting expansion and splitting of the initial macro-concepts into finer-grained operational definitions while retaining the dimension framework. For each LOGEXP, we computed the number of documents in which the LOGEXP Boolean expression matched in the title or abstract (nDocs) and the mean embedding vector across all documents matching that LOGEXP. Pairwise Euclidean distances between LOGEXP embedding vectors yielded a 49 × 49 distance matrix summarizing semantic proximity among the concepts.

*Similarity and clustering.* Hierarchical clustering with average linkage (UPGMA: Unweighted Pair Group Method with Arithmetic Mean) was applied to obtain a dendrogram of semantic relationships among the LOGEXPs.

*Low coverage concept identification.* We defined low coverage concepts, which can be considered proxies to represent gaps in the literature, based on the distribution of the *nDocs* values (number of documents matching the LOGEXP query) across the 49 LOGEXPs (Supplementary Figure S1c). The 15^th^ percentile has been identified as a cut-off to define low coverage concepts, and corresponds to an *nDoc*s ≤ 20, which includes 7 out of 49 concepts (14.3%).

## Results

**Figure 1.**
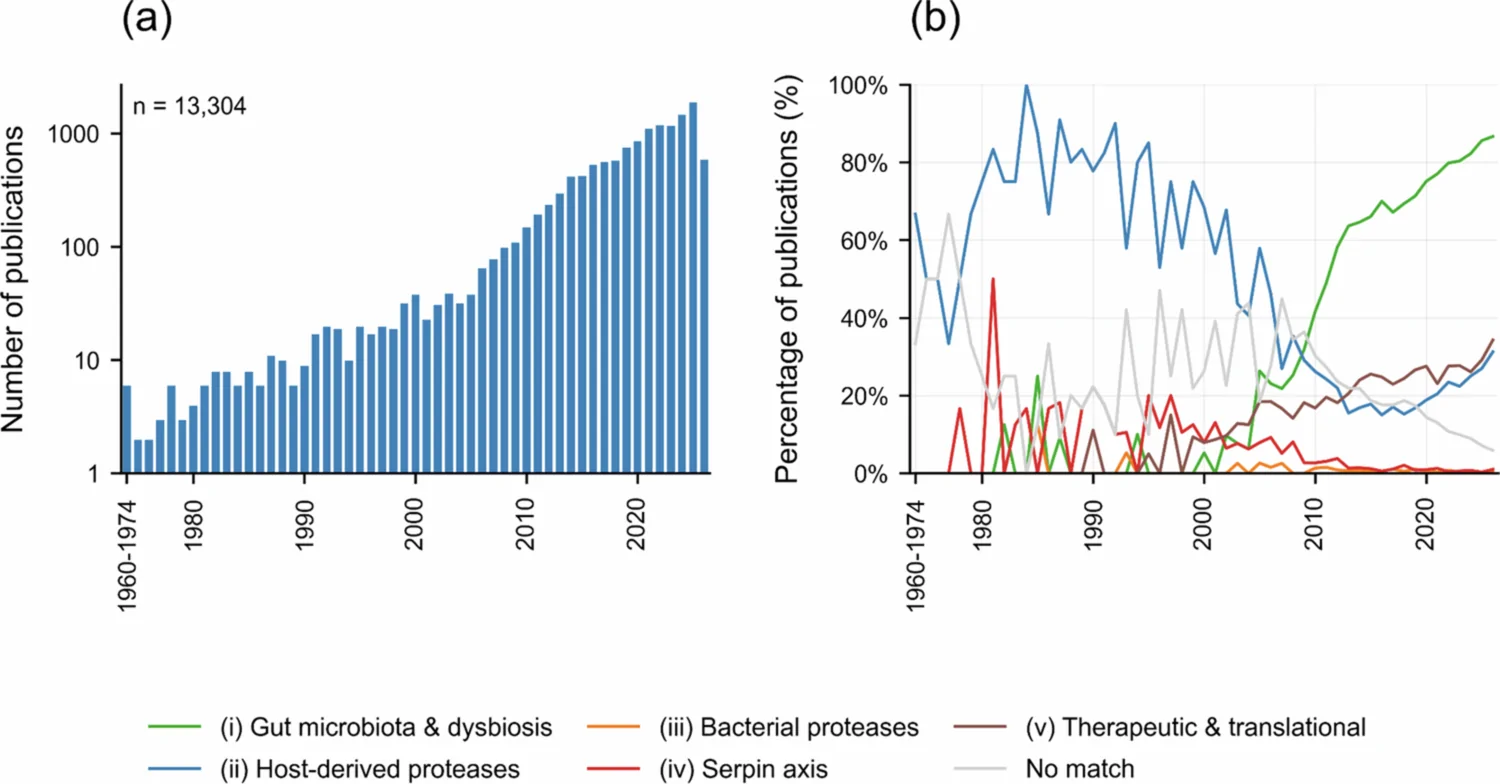
IBD-related research has shifted over decades towards greater emphasis on gut microbial ecology, host proteolytic regulation, and therapeutic translation. a. Number of PubMed-indexed publications in the corpus along the horizontal timeline. b. For each time interval, each colored curve shows the percentage of corpus articles whose abstracts contain at least one term from the dimension-specific vocabulary for the corresponding dimension (percentages are relative to the corpus articles published in the specific year, Supplementary Table ST2). Dimensions (i–v) are not mutually exclusive. The gray curve indicates articles with no abstract vocabulary match in dimensions (Supplementary Table ST3).

We applied a text mining approach to map the conceptual landscape of IBD research across the protease-serpin-microbiome axis from 13,304 published manuscripts (Figure 1a, Methods). We identified 31 macro-thematic groups and consolidated them into five dimensions, representing the main axes of the protease-serpin-microbiome in IBD: *(i)* gut microbiota and dysbiosis; *(ii)* host-derived proteases; *(iii)* bacterial proteases; *(iv)* serpin axis; and *(v)* therapeutic and translational aspects (Supplementary Figure S2, Supplementary Material S1, Supplementary Table ST1, Methods). IBD research has progressively moved throughout these different dimensions, however, they represent biological interconnected axes of intestinal proteolytic homeostasis. Despite this biological connection, these dimensions are often represented as separate concepts, raising the question of whether host and microbial proteolytic activity have been considered in the context of IBD, and whether their integration may improve our understanding of proteolytic imbalance, epithelial dysfunction, inflammation, and disease progression.

### Landscape of protease-serpin in IBD

Between 1960 and 1990, the indexed literature in the corpus was predominantly focused on the ‘host-derived proteases’ (dimension *(ii)*), with the ‘serpin axis’ (dimension *(iv)*) representing the second most-important dimension (Figure 1b). This early dominance reflects a period in which host-derived proteases, circulating antiproteases, acute-phase proteins, and neutrophil-associated proteolysis represented accessible molecular markers of intestinal inflammation in IBD.^73-76^ The ‘gut microbiota and dysbiosis’ (dimension *(i)*) was already detectable from the 1980s, but remained comparatively sparse relative to ‘serpin axis’ (dimension *(iv)*) and ‘host-derived proteases’ (dimension *(ii)*), suggesting that microbial contributions were not yet a central feature of IBD research in that period (Figure 1b).

The ‘bacterial proteases’ (dimension *(iii)*) first appeared in 1985, with sparse retrievals throughout the 1990s coincide with growing attention to microbial virulence factors and host-microbe interactions.^77^ Among the articles published during this period, only one matched the bacterial protease vocabulary (*dimension (iii)*), whereas 134 and 25 were associated with host protease and serpin/antiprotease vocabularies (*dimensions (ii)* and *(iv)*), respectively (Figure 1b and Supplementary Table ST3). Representative examples from this period included studies on fecal proteinase activity, as well as investigations of inflammatory proteolytic pathway.^78-80^ From the 2000s, ‘gut microbiota and dysbiosis’ (dimension *(i)*) gained momentum, driven by increasing genomic and metagenomic sequencing of the human gut microbiome.^81-83^ Conversely, serpin axis studies declined after 2010, carrying less relative weight recently than research on, host proteases, and translational themes. These trends could suggest that serpin became integrated and investigated as a regulatory component within broader inflammatory, epithelial, or microbiome-related contexts rather than as a central framework for understanding intestinal proteolytic regulation. Finally, ‘therapeutic and translational’ research (dimension *(v)*) showed an increasing trend after 2012, consistent with a period in which biological and targeted therapies attracted the interest of the research communities towards progressive translation of mechanistic insights into treatment-oriented study designs.^84-86^

#### Corpus-level concept coverage and cross-domain connectivity

**Figure 2.**
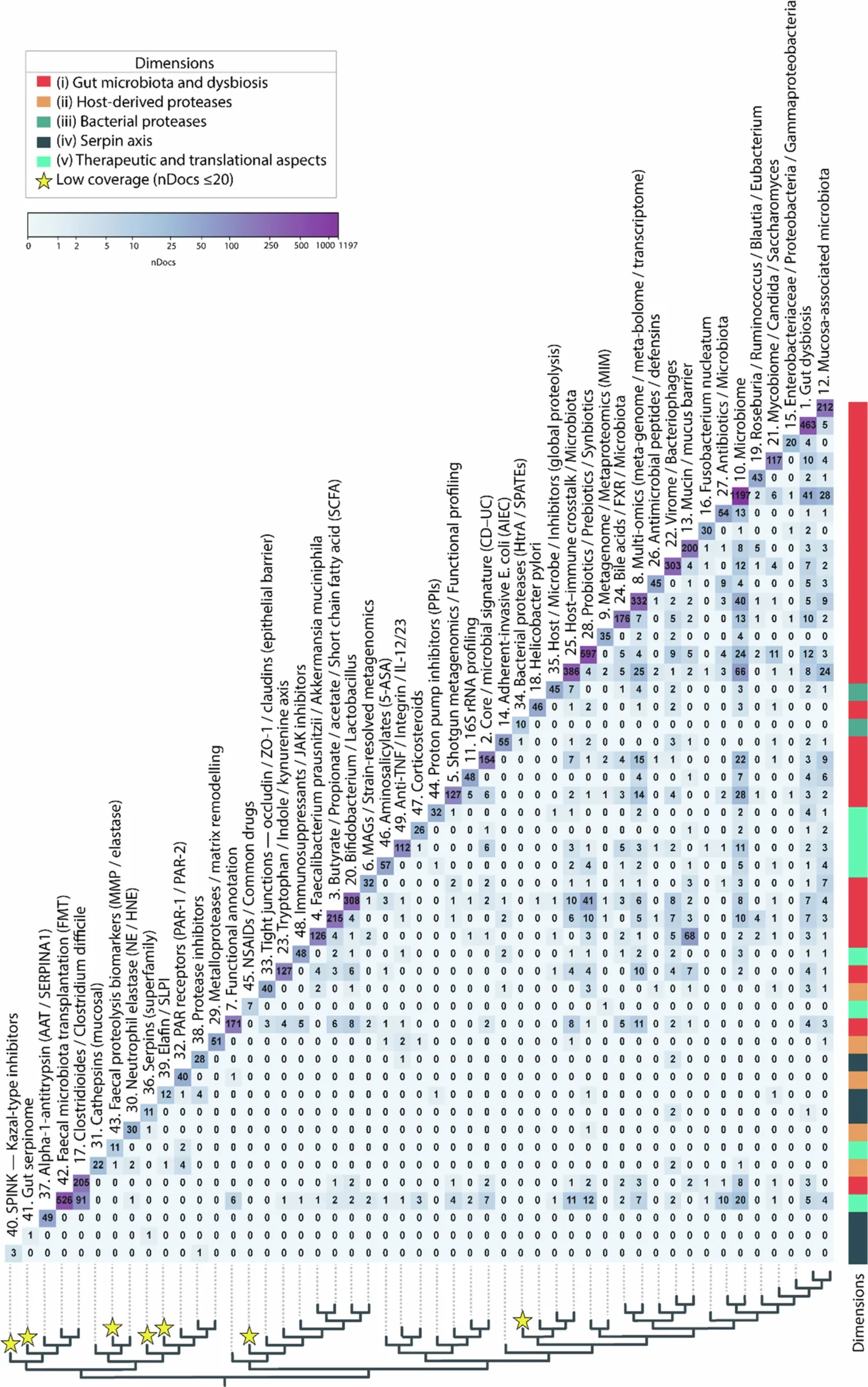
Pairwise LOGEXP joint retrieval reveals a fragmented conceptual landscape dominated by a microbiome-host-immune core. The cladogram represents the hierarchical clustering of concepts according to semantic similarity in embedding space (UPGMA; Supplementary Figure S4). The lower-triangular heatmap displays concepts co-occurence, with each cell reporting the number of corpus shared by the corresponding concepts. Diagonal numbers represent the number of documents for the single concept.

We evaluated concept-level coverage and integration across the protease-serpin- axis using 49 LOGEXPs derived from the macro-thematic structure of the enriched-term dendrogram (Supplementary Figure S2 and Supplementary Table ST1). The five strongest joint retrieval were: *Clostridioides/Clostridium difficile* and *Fecal microbiota transportation FMT* (*n* = 91), *Faecalibacterium prausnitzii/Akkermansia muciniphila* and *Mucin/mucus barrier* (*n* = 68), *Microbiome* and *host-microbiota-immune crosstalk* (*n* = 66), *Gut dysbiosis* and *Microbiome* (*n* = 41), *Bifidobacterium/Lactobacillus-Probiotics* and *Prebiotics/Synbiotics* (*n* = 41) (Figure 2). This pattern shows that the most interconnected areas of the IBD microbiome literature are centered around clinically established microbial signatures, barrier-associated taxa, and -targeted interventions. In contrast, seven LOGEXPs were lowly covered: *gut serpinome* (*n* = 1), *SPINK* (*n* = 3), *NSAIDs/common drugs* (*n* = 7), *bacterial proteases (HtrA/SPATEs)* (*n* = 10), *serpins (superfamily)* (*n* = 11), *fecal proteolysis biomarkers* (*n* = 11), and *elafin/SLPI* (*n* = 12) (Supplementary Figures S3 and S4, Supplementary Table ST1, Methods). Except for *NSAIDs*, the other concepts were relevant components of the protease-serpin- axis, but less frequently retrieved in titles and abstracts, with very modest joint retrieval with the -host-immune hubs (Supplementary Figures S4 and S5). We interpret this limited coverage as a constraint of the semantic representation of these concepts, rather than an indication of their biological significance.

### Integrating the protease-serpin axis

#### Host-derived proteases: inflammatory signaling and tissue remodeling

**Figure 3.**
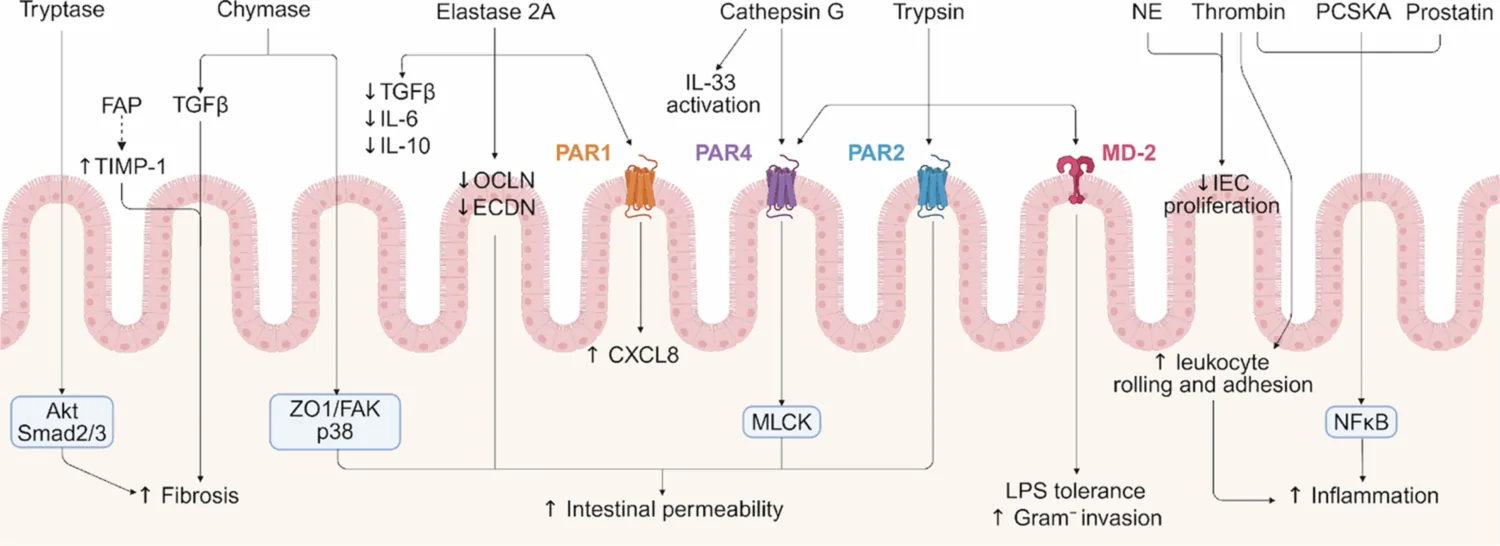
Overactive host-derived serine proteases exert a pro-inflammatory effect on the gut. The molecular mechanisms and functions of overactive host-derived serine proteases in gut inflammation, including intestinal permeability disruption and immune response. Akt, protein kinase B; CXCL, chemokine (C-X-C motif) ligand; ECDN, E-Cadherin; FAK, focal adhesion kinase; FAP, fibroblast activation protein; IEC, intestinal epithelial cell; IL, interleukin; MLCK, myosin light-chain kinase; OCLN, occludin; PAR, protease-activated receptor; TGFβ, transforming growth factor beta; TIMP-1, tissue inhibitor of metalloproteinases 1; ZO1, zonula occludens-1.

*Increased host protease activity in IBD*. Several host-derived serine proteases show increased activity in the intestinal mucosa or feces of IBD patients, including trypsin-like proteases, mast cell tryptase and chymase, elastase 2A, human neutrophil elastase, proteinase 3, cathepsin G, and thrombin.^6^ ^,^ ^61^ ^,^ ^64^ ^,^ ^87^ Conversely, HtrA1, HtrA2, protein C, and matriptase, may show reduced activity.^88^ These changes affect the gut through three interconnected mechanisms (Figure 3).

*Barrier integrity*. Several proteases act directly on epithelial junctions. Mast cell chymase disrupts tight and adherens junctions via the ZO-1/FAK/p38 axis^88^ ^,^ ^89^; elastase 2A lowers occludin and E-cadherin expression^6^ ^,^ ^90^; and trypsin-like activity, acting partly through Par-2/PAR/4, increases intestinal permeability.^91-93^ Neutrophil elastase and thrombin additionally impair epithelial cell proliferation.^7^ ^,^ ^91^ This loss of proliferative capacity reflects a metabolic and renewal dimension of epithelial regulation, alongside the structural and signaling effects described above.

*Immune modulation*. These proteases also shape mucosal immune signaling. Trypsin cleaves the TLR4 co-receptor MD2, promoting Gram-negative bacterial invasion,^61^ ^,^ ^91^ ^,^ ^94^ while thrombin enhances leukocyte adhesion to the endothelium.^87^ ^,^ ^95^ Several proteases directly regulate cytokine and chemokine output: trypsin induces CXCL8^91^; elastase 2 A lowers TGF-*β*, IL-6, and IL-10 while raising CXCL8^64^; cathepsin G activates IL-33^96^; and plasmin promotes MMP-9-mediated cytokine release.^97^ Thrombin, prostasin, and PCSKA can also activate NF-κB signaling, independent of cytokine processing.^95^ ^,^ ^98^ Much of this immune signaling converges on protease-activated receptors: elastase 2 A signals through PAR-1,^92^ ^,^ ^93^ cathepsin G and tryptase through other PARs,^91^ ^,^ ^99^ ^,^ ^100^ and PAR-1/-3/-4 activation overall drives apoptosis, permeability, and leukocyte adhesion.^101^ ^,^ ^102^

*Tissue remodeling and coagulation*. Proteases also remodel the extracellular matrix (ECM), contributing to IBD-associated fibrosis.^103^ Granzyme A and uPA degrade non-fibrotic tissue, whereas chymase activates MMP-9 and tryptase drives fibroblast-to-myofibroblast differentiation via Akt/Smad2/3 signaling^104^ ^,^ ^105^; chymase-mediated TGF-*β* activation and FAP-dependent TIMP-1 production further promote fibrosis.^12^ Coagulation is similarly disrupted: elevated thrombin and uPA activity, combined with reduced protein C and tPA activity, create a procoagulant state that impairs regulation of colitogenic CD4⁺ T cells and disrupts fibrinolysis.^106-109^

Our text-mining analysis suggests that these are often explored as distinct axes within IBD, *PAR receptors*, *tight junctions*, *neutrophil elastase*, and *serpins* comprised only four, three, two, and two shared documents, respectively, in contrast to the dominant *Clostridioides/Clostridium difficile* and *Fecal microbiota transplantation (FMT)* core (*n* = 91), Figure 2 and Supplementary Figure S3). This suggests IBD pathogenesis involves interconnected but differentially emphasized biological layers, whose integration remains essential for a systems-level understanding of the disease.

#### Bacterial proteases

**Figure 4.**
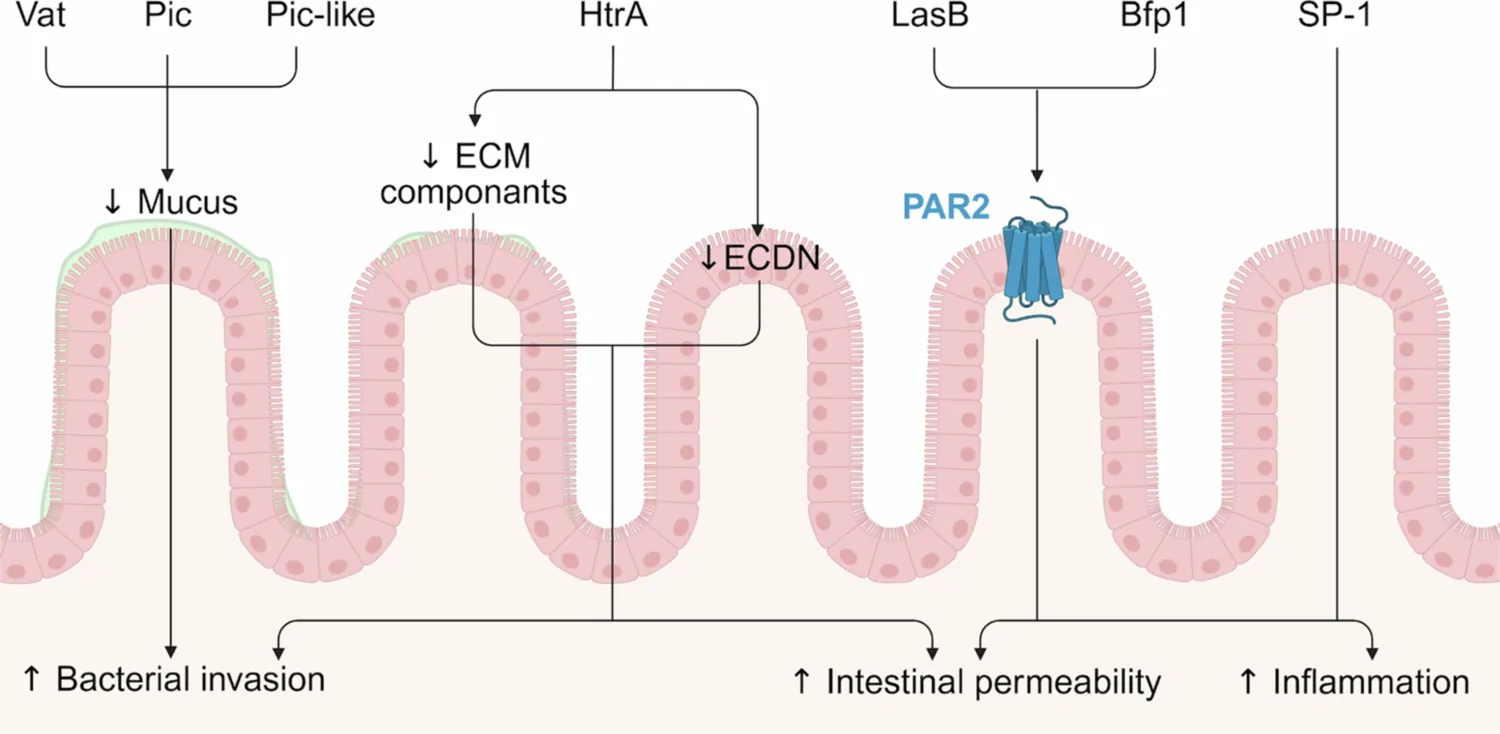
Microbial serine proteases are involved in gut barrier disruption and inflammation. The pathogenic mechanisms of microbial serine proteases in IBD. ECDN, E-Cadherin; ECM, extracellular matrix; HtrA, high-temperature requirement A; PAR, protease-activated receptor.

Despite their potential relevance, the contribution of bacterial serine proteases to gut proteolytic activity remains poorly characterized, especially in the context of IBD.^5^ ^,^ ^110^ The high-temperature requirement A (HtrA) bacterial serine proteases has been reported to be increased in IBD compared to healthy individuals.^111^ HtrA proteases have a role in regulating microbial pathogenesis and inflammation,^112^ ^,^ ^113^ they can facilitate bacterial colonization and invasion, as shown in pathogens such as *Bacillus anthracis*, *Borrelia burgdorferi*, *Campylobacter jejuni*, and *Helicobacter pylori* ^114-117^, and their disruption, observed in infections with *C. jejuni*, *H. pylori*, and *E. coli*, weakens barrier integrity and is a hallmark of IBD^118-122^ (Figure 4). Proteases like Vat, Pic, and Pic-like SPATEs, produced by taxa such as *Shigella*, *Neisseria*, *Citrobacter*, and several *E. coli* pathotypes, can degrade the protective mucus layer, enhancing bacterial colonization, while Sat induces junctional damage in cultured intestinal epithelial cells.^123-128^ SPATE-encoding *E. coli* strains are frequently isolated from IBD tissues, and their ability to degrade intracellular defensins further supports a role in disease progression^129^ ^,^ ^130^ (Figure 4). Other mechanisms include *H. pylori* trypsin-like enzymes activating PAR-2 in gastric epithelium, and *Pseudomonas aeruginosa* LasB elastase cleaving PAR-2 in inflamed duodenal tissue.^14^ Epithelial PAR signaling is implicated in IBD pathophysiology in response to proteases from *Enterococcus faecalis*, *Porphyromonas gingivalis*, and *Clostridium difficile* ^131-134^. Also non-pathogenic bacteria, like *Bacillus subtilis*, can affect host homeostasis via serine protease activity, contributing to the procoagulant state via human prothrombin often observed in IBD.^135^ ^,^ ^136^ Recent studies also highlighted newly characterized bacterial proteases from an unclassified *Clostridium* species, to increase epithelial permeability, disrupts microbial composition, and exacerbates inflammation in a DSS-induced mouse model of colitis.^137^ ^,^ ^138^ Elevated serine protease activity in the IBD gut, regardless of whether it originates from the host or the microbiome, plays a central role in driving barrier disruption, immune activation, and ongoing inflammation. The diverse mechanisms of these enzymes and their impact on host physiology underscore the critical need to further investigate microbial proteases as viable therapeutic targets.

#### Serpins and endogenous inhibitors

Intestinal proteolytic homeostasis relies on a balanced interplay between proteases and their endogenous inhibitors. In IBD, this equilibrium is frequently disrupted, with serine protease inhibitors (serpins), produced by both host tissues and the, playing a critical, yet often dysregulated, role in controlling inflammation, tissue integrity, and immune signaling.

Serpins are the largest protease inhibitor superfamily, conserved across all kingdoms of life.^139-141^ These 350-400 amino acid proteins share a conserved structure with three *β*-sheets and seven to nine *α*-helices.^139^ Divided into 16 clades, they inhibit serine and papain-like cysteine proteases and caspases, while also acting as chaperones, antiangiogenic factors, and hormone transporters.^139^ ^,^ ^140^ ^,^ ^142-148^ In humans, 30 of 37 serpins primarily act as protease inhibitors involved in tissue remodeling, coagulation, and inflammation resolution.^127^ ^,^ ^140^ ^,^ ^141^ ^,^ ^149-151^ Several mucosal serpins are decreased in IBD patients: downregulated kallistatin can cause unchecked colitogenic kallikrein activity,^152^ reduced Tumor-Associated Trypsin Inhibitor (TATI) breaks down protease control,^153^ ^,^ ^154^ and depleted antithrombin III impairs inflammation attenuation via NF-κB modulation.^152^ ^,^ ^155^

Certain serpins are upregulated by inflammation, reflecting dynamic gut regulation. For instance, mucosal SerpinA3N increases in IBD but fails to fully neutralize neutrophil elastase, and its depletion worsens mouse colitis.^11^ ^,^ ^154^ AAT, produced by the liver, epithelial, and immune cells, rises during inflammation to inhibit neutrophil elastase, proteinase 3, cathepsin G, and trypsin.^156-158^ AAT deficiency correlates with poor UC outcomes,^159^ and fecal AAT drops in IBD.^159^ ^,^ ^160^ Notably, AAT supplementation restores barrier integrity and reduces inflammation in colitic mice.^8^ In pediatric patients, fecal AAT serves as a non-invasive inflammatory marker, though it is less specific than calprotectin.^161^

SERPINE1 (PAI-1) promotes fibroblast activation and intestinal fibrosis via IL-33 induction in Crohn’s-like ileitis models.^162^ Its single-cell RNA-seq-verified upregulation in inflamed mesenchymal cells highlights the IL-33-SERPINE1 axis as a therapeutic target in fibro-stenosing IBD. Conversely, SERPINEB1 is highly expressed in UC mucosa and experimental colitis, protecting the mucosa by inhibiting neutrophil elastase and cathepsins.^163^ ^,^ ^164^ Similarly, epithelial and immune-derived secretory leukocyte protease inhibitors (SLPI) limit neutrophil elastase, mast cell proteases, and pro-inflammatory cytokines like TNFα and CXCL8.^165^ SLPI is elevated in UC lesions and dampens inflammation in experimental colitis.^9^ ^,^ ^165^ ^,^ ^166^

SPINK4, a goblet cell-specific inhibitor, is a novel IBD biomarker and regulator. Upregulated in UC mucosa, it promotes mucus layer regeneration and goblet cell differentiation. Its secretion is stimulated by the gut, serving as a host-microbe interface modulator and a non-invasive disease activity marker.^167^ Trappin-2 (elafin), another epithelial inhibitor, contains a protease-inhibitory elafin domain and a matrix-binding domain.^165^ ^,^ ^166^ Elafin expression decreases in IBD tissues, correlating with elevated protease activity and inflammation.^81^ Beyond direct inhibition, elafin provides antimicrobial and anti-inflammatory effects, and its upregulation improves outcomes in colitis models.^10^ ^,^ ^168^ Notably, *Lactobacillus plantarum* induces Trappin-2 expression via TLR9 signaling, suggesting probiotic therapeutic potential.^169^ Engineered probiotics overexpressing elafin demonstrate superior efficacy to IL-10 or TGF-*β* in reducing inflammation and restoring barrier function.^166^ ^,^ ^169^

#### Prokaryotic serpins and the gut serpinome

Prokaryotic serpins occur in approximately 13% of bacteria and 31% of sequenced archaeal genomes.^125^ ^,^ ^126^ Their *in vivo* functions remain unknown, but *in vitro* studies confirm their inhibitory potential. Extremophilic serpins, such as those from *Perabaculum neutrophilum*, display robust structural adaptations by resisting misfolding and remaining stable at high temperatures.^123^ First reported in 2002 by Irving and colleagues across 12 archaeal and extremophilic sequences,^170^ their presence in host-associated niches like the gut or oral cavity initially suggested an evolutionary origin via horizontal gene transfer from eukaryotes.^126^ ^,^ ^129^ ^,^ ^130^ ^,^ ^170^ However, the discovery of serpins in free-living soil and marine bacteria indicates that they form an ancient protein superfamily that evolved in prokaryotes before diversifying across kingdoms.^170^ This is supported by phylogenetic analyzes of over 6,000 sequences, which classify microbial serpins into groups T and U, many with predicted inhibitory functions.^169^ ^,^ ^170^

Several prokaryotic serpins have been characterized, including thermopin from *Thermobifida fusca*, a biotechnology-relevant chymotrypsin inhibitor stable at 60 °C which forms stable covalent complexes with its target.^124^ ^,^ ^170^ Tengpin, produced by *Thermoanaerobacter tengcongensis*, belongs to the T group and covalently binds neutrophil elastase.^171^ ^,^ ^172^ In *Clostridium thermocellum*, PinA protects the cellulose-degrading cellulosome from subtilisin-like proteases,^173^ ^,^ ^174^ while a second serpin provides cross-class inhibition against both serine and cysteine proteases like papain.^175^ Additionally, vioserpin from *Gloeobacter violaceus* shares human-like features such as heparin-binding and trypsin-like protease inhibition, whereas sponge-microbe metagenomic PI-QT inhibits trypsin and alpha-1-antichymotrypsin.^175-177^ Lastly, *Tannerella forsythia* secretes Miropin, a highly flexible, broad-spectrum serpin with multiple reactive center loop cleavage sites that targets both microbial and host proteases (including neutrophil elastase, cathepsin G, trypsin, and human plasmin), potentially enhancing virulence and bacterial persistence in host tissues.^14^ ^,^ ^129^ ^,^ ^131^

Most characterized prokaryotic serpins are not native to the gastrointestinal tract, limiting their relevance to IBD. However, emerging data on gut-associated microbial serpins, or the gut serpinome,^72^ suggest a novel mechanism of intestinal homeostasis. Characterized gut serpins include those from *Bifidobacterium longum*, *Eubacterium siraeum*, and *Eubacterium saburreum*. In *B. longum*, serpin genes promote survival in the hostile gut environment.^133-135^ Its SerpinBL potently inhibits neutrophil elastase, forms stable complexes with fecal proteases in mice, and protects the intestinal mucosa,^178^ ^,^ ^179^ modulating host protease activity, preserving barrier integrity, and reducing inflammatory signaling. SerpinBL also reduces enteric neuron activation in irritable bowel syndrome to alleviate pain and counteracts gliadin-induced inflammation, suggesting applications in celiac disease.^180^ ^,^ ^181^ *Eubacterium saburreum* produces saburopein, which inhibits luminal pancreatic elastase.^182^ *Eubacterium siraeum* produces siropins 1 and 2, which inhibit neutrophil elastase and proteinase 3, marking the first microbial serpins to inhibit human proteinase 3 and reduce fecal elastase-like activity in DSS-induced colitis.^183^ Additionally, parasites like *Trichinella spiralis* secrete serpins that inhibit serine and cysteine proteases.^184^ ^,^ ^185^ These insights reinforce that serpin-mediated proteolytic regulation is a conserved, critical mechanism across diverse gut symbionts and pathogens.

Collectively, the lack of an integrated “gut serpinome” framework in the literature, despite evidence for individual serpins, underscores a reliance on fragmented terminology. Advancing our understanding of this regulatory network will require both targeted mechanistic studies and harmonized reporting to effectively bridge microbial inhibitory pathways with host mucosal protease dynamics in IBD.

## Discussion

Although literature often conceptually separates host proteases, microbial proteases, and inhibitory pathways, these systems interact biologically at the intestinal interface. Rather than indicating absent interactions, reduced co-retrieval in text-mining reflects terminology and indexing constraints. To address this, we integrate these mechanisms into a proteolytic homeostasis perspective where the protease-serpin- axis converges to regulate epithelial integrity, mucosal immunity, and tissue remodeling in IBD.

Experimental studies support individual roles for host and microbial proteases, but direct evidence of their cooperative or synergistic action during IBD progression remains limited. For instance, while bacterial HtrA proteases target cadherins and neutrophil elastase affects claudins and extracellular-matrix components,^6^ ^,^ ^118^ their combined impact is unknown. Investigating these overlapping activities and their interactions with genetic factors, such as AAT deficiency, represents a critical future direction.

Characterizing poorly understood components like SPATEs and microbial serpins remains a key priority. Functional studies are needed to clarify their roles in intestinal proteolytic regulation and host inhibitory pathways. These research gaps must be evaluated alongside the limitations of abstract-based text-mining, which may overlook relevant data described with alternative terminology or contained exclusively within full-text articles.

Therapeutic interventions targeting this axis show varying stages of development. Selective neutrophil elastase inhibitors like sivelestat reduce colitis severity in rodents, while AZD9668 has reached phase II trials for COPD^186^ but remains untested in IBD. Conversely, AAT supplementation via Prolastin and Zemaira has demonstrated pilot efficacy in small cohorts.^8^ Future research should map strain-level protease and serpin expression in patient microbiomes to evaluate host-microbial interactions and biomarker potential. While microbiome-targeted strategies like engineered probiotics expressing protective serpins or modulating specific taxa are promising, they currently lack direct clinical validation in IBD.

## Conclusions and future perspectives

Our text-mining analysis of the protease-serpin- axis reveals a striking disconnect between the biological reality of intestinal homeostasis and its representation in IBD literature. While host proteases, microbial proteolytic activity, and endogenous serpins are interdependent drivers of epithelial integrity, mucosal immunity, and tissue remodeling, they are frequently investigated as isolated research domains. This conceptual fragmentation—where modules such as barrier proteolysis, neutrophil elastase, and PAR signaling operate in semantic silos, likely reflects historical research divisions rather than biological separation.

To overcome this, we propose an integrated approach that positions the protease-serpin- axis as a unified regulatory network. By synthesizing these components, we gain a systems-level view of IBD pathogenesis, highlighting critical opportunities for innovation. Translational strategies supported by current evidence, such as selective protease inhibition and AAT-based therapies, warrant prioritized investigation, while emerging microbiome-based interventions—including engineered probiotics designed to restore protective serpin levels—represent a promising frontier. Moving forward, harmonizing terminology and reporting standards in the literature will be crucial in bridging these domains.

## Supplementary Material

Supplementary Material AlmakiM_DePerriCR_etal__rev1_SUPPLEMENTARY_TABLES.xlsx

Supplementary Material AlmakiM_DePerriCR_etal__rev1_SUPPLEMENTARY.docx

## Data Availability

The scripts developed and used in this work, together with the text mining data are available at doi: 10.5281/zenodo.20448008.
