# Supplementary material for: Protease-serpin-microbiome interactions in inflammatory bowel disease: toward integrated biomarkers of gut health
Source: Gut Microbes. 2026 Sep 2;18(1):2726641. doi: 10.1080/19490976.2026.2726641 (PMC13540094; doi:10.1080/19490976.2026.2726641)
Supplement: Supplementary file 1 [file KGMI_A_2726641_SM2497.docx]

**Protease-Serpin-Microbiome Interactions in Inflammatory Bowel Disease: Toward Integrated Biomarkers of Gut Health**

Meshal Almalki ^a,+^, Camilla R. De Pierri ^b,+^, Tristan Méric ^a^, Héla Mkaouar ^a^, Donia Slimani ^a^, Vincent Mariaule ^a^, Sirine Tebassi ^a^, Nizar Akermi ^a^, Emmanuelle Maguin ^a^, Juan Hernandez ^a,c^, Roberto T. Raittz ^d^, Francesco Asnicar ^b,^*, Moez Rhimi ^a,^*

*^a^ Université Paris-Saclay, INRAE, AgroParisTech, Micalis Institute, MIHA Team, Jouy-en-Josas, France*

*^b^ Department of Cellular, Computational and Integrative Biology, University of Trento, Trento, Italy*

*^c^ Oniris VetAgroBio Nantes, Nantes, France*

*^d^ Laboratory of Artificial Intelligence Applied to Bioinformatics, Universidade Federal do Paraná, Curitiba, Brazil*

^+^ Equal contribution

* Corresponding author: Francesco Asnicar (f.asnicar@unitn.it), Moez Rhimi (moez.rhimi@inrae.fr)

**Supplementary Material**

Note: The dimensions provide the high-level thematic scaffold, while macro-groups represent groupings derived from the literature associated with each keyword/node. For each word in the global tree, we inspected the corresponding word output (Words.html) and reviewed the set of linked articles/abstracts captured by the embeddings around that term. Macro-group assignments were then made by synthesising the dominant ideas and recurring biological/technical terms across those abstracts

**Supplementary Material S1.** Thematic dimensions and macro-groups: concept-informed background (IBD literature)

# **i. Gut microbiome & dysbiosis**

**Composition/imbalance:** Centres on gut dysbiosis as a higher-order descriptor of altered community structure and function in IBD. Literature in this vein links shifts in taxonomic and functional profiles to inflammatory activity, relapse risk, and treatment exposure, often integrating host multi-omics readouts from patient cohorts [1,3,34]. Closely related work seeks reproducible core-microbiome patterns and microbial signatures that differentiate Crohn’s disease from ulcerative colitis beyond generic dysbiosis descriptors [35,36].

**Microbial metabolites:** The emphasis is on short-chain fatty acids, especially butyrate, propionate, and acetate, which serve as microbial fermentation products that signal to the epithelium and mucosal immune compartment. SCFAs are widely discussed as mediators of barrier support, regulatory tone, and metabolic crosstalk between diet, microbiome, and host [4,35].

**Indicator taxa:** Highlights *Faecalibacterium prausnitzii* and *Akkermansia muciniphila* as taxa repeatedly invoked as ecological indicators: the former tied to butyrate-producing potential and anti-inflammatory folklore in cohort studies, the latter to mucin-foraging behaviour and mucosal interface ecology [1,3,35]. Together, they exemplify how specific lineages operationalise “dysbiosis” beyond generic richness metrics

**Metagenomics:** Shotgun and whole-metagenome profiling, strain-resolved analysis via MAGs, functional annotation of pathways, and multi-omics coupling of metagenomes to metabolome, metatranscriptome, and metaproteome. It also covers 16S rRNA profiling as a taxonomic survey layer, as well as the broader intestinal microbiome literature, in which sequencing depth and functional inference are central.

**Spatial niche:** Mucosa-associated microbiome versus luminal communities: different oxygen tension, immunoglobulin coating, and proximity to the epithelium create distinct niches that shape which organisms participate in mucosal inflammation and healing [1,35]. The mucus layer and mucin barrier form a further spatial axis at this interface, governing microbial access to the epithelium and shaping mucosal colonisation niches [18].

**Pathobiont:** Adherent-invasive *E. coli* (AIEC) and expansions of *Enterobacteriaceae*, *Proteobacteria*, *Gammaproteobacteria* embody pathobiont and bloom narratives in IBD—adhesion, invasion, macrophage survival for AIEC, and Proteobacteria-rich states linked to inflammation and post-antibiotic ecology [9,10]. Named pathobionts discussed in IBD mucosa also include *Fusobacterium nucleatum*, *Clostridioides difficile*, and *Helicobacter pylori* [37–39].

**Firmicutes:** *Roseburia, Ruminococcus*, *Blautia*, and *Eubacterium*, within the *Firmicutes*-rich fibre-fermenting guild, are often contrasted with dysbiotic signatures in IBD cohorts [1,3].

**Commensals:** *Bifidobacterium* and *Lactobacillus* (and related *Lactobacilli*) capture classical commensal and probiotic-associated lineages discussed in relation to competitive exclusion, fermentation, and immune conditioning at the mucosa [29,32].

**Multi-kingdom:** The mycobiome (*Candida*, *Saccharomyces*) and the virome (bacteriophages, phage taxa such as *Caudovirales*) extend IBD ecology beyond bacteria, with emerging work on fungal-bacterial interactions and phage dynamics during inflammation [20,21].

**Microbial-immune metabolites:** The tryptophan-indole and kynurenine axis summarises microbial metabolism of tryptophan into bioactive ligands that engage host immune programmes (including AhR-linked pathways and innate lymphoid circuits), bridging diet-microbiome-immunity in mucosal disease [5,6].

**Microbial metabolism:** Bile acids and their microbial biotransformation, together with nuclear receptor signalling such as FXR and the microbiome-bile acid axis, frame how co-metabolism reshapes epithelial and immune responses beyond classical SCFA narratives [7,8,1].

**Mechanism:** Host-immune crosstalk with the microbiome foregrounds regulated circuits (pattern recognition, cytokine networks, innate and adaptive coordination) through which microbial signals are interpreted as homeostatic or inflammatory at the mucosa [1,34]. Antimicrobial peptides and defensins sit within this host-microbe defence layer as epithelial effectors that shape microbial community structure at the barrier [18,33].

**Iatrogeny:** Antibiotics and antimicrobial exposure targeting the microbiome are treated as ecological shocks, with epidemiological links to IBD onset and flares and experimental models of *Enterobacteriaceae* expansion underpinning this theme [26,27,28].

**Dietary modulation:** Probiotics, prebiotics, and synbiotics capture dietary and live-biotherapeutic strategies aimed at reshaping fermentation output, colonisation resistance, and mucosal immune tone as adjuncts to drug therapy [32].

# **ii. Host-derived proteases**

**Metalloproteases:** Matrix metalloproteinases (MMP family) and TIMP-mediated regulation epitomise matrix remodelling in inflamed intestine: stromal digestion, angiogenesis, and fibrotic remodelling are recurrent motifs in IBD mucosa [11,12].

**Serine protease (innate):** Neutrophil elastase (NE/HNE) drives innate serine protease-mediated tissue injury and amplifies inflammatory cascades in active disease [2,13].

**Cysteine proteases:** Cathepsins at the mucosal interface participate in lysosomal processing, matrix turnover, and cell death programmes that accompany chronic inflammation [2].

**Epithelial signalling:** PAR-1 and PAR-2 (protease-activated receptors) translate protease activity in the lumen and leukocytes into epithelial secretion, chemokine output, and neuroimmune signalling [2,17].

**Epithelial barrier/tight junctions:** Tight junction proteins-occludin, ZO-1 (and ZO-2/3), claudins, MLCK signalling—encode epithelial barrier competence; their protease- and cytokine-sensitive regulation is central to permeability debates in IBD [2,16,18].

# **iii. Bacterial proteases**

**Microbial virulence:** Enteric bacterial proteases, such as HtrA and SPATE-autotransporter proteases, exemplify factors that cleave mucins, junctional proteins, or immune mediators, promoting adherence and mucosal stress [2,9].

**Global Proteolysis**: The host-microbe-inhibitor nexus and global proteolysis frame mucosal proteolysis as the net outcome of overlapping host, immune, and microbial proteases and their inhibitors, relevant to barrier function and visceral sensitivity in intestinal disease [2,13].

# **iv. Serpin axis**

**Serpin/antiprotease:** The serpin superfamily provides endogenous inhibition of serine proteases via a conserved conformational trap, contextualised in mucosal inflammation as a brake on unchecked proteolytic cascades [2,15].

**Circulating inhibitor/genetics:** Alpha-1-antitrypsin (AAT, SERPINA1) links circulating antiprotease capacity and genetic deficiency syndromes to the systemic protease-antiprotease balance and its intersection with intestinal inflammation [15].

**Broad class:** The protease inhibitor literature—especially serine protease inhibitors as a broad pharmacological and endogenous class—intersects coagulation, complement, and inflammatory cascades co-activated in IBD [2,15].

**Epithelial/leukocyte antiproteases:** Elafin and SLPI (secretory leukocyte protease inhibitor) are epithelial- and leukocyte-derived antiproteases that defend the surface mucosa during infiltrative inflammation [15].

**Kazal family:** SPINK (Kazal-type) inhibitors, including SPINK4, represent a modular antiprotease family discussed in mucosal repair and combination with anti-inflammatory biologics in experimental colitis [14,15].

**Umbrella term/serpinome:** The intestinal serpinome is used as an umbrella construct for co-regulated serine-protease inhibitors in the gut environment, integrating host and (increasingly) microbiome-derived inhibitors into a single mucosal protease-antiprotease narrative.

# **v. Therapeutic & translational**

**Clinical microbiome intervention:** Faecal microbiome transplantation (FMT) encapsulates attempts to reset ecosystem function and composition in ulcerative colitis and related indications, with heterogeneous RCT outcomes [22,1].

**Biomarkers:** Faecal proteolysis biomarkers—MMP and elastase readouts in stool—sit alongside classical inflammatory markers as translational endpoints linking luminal protease activity to disease activity [19,2].

**Conventional therapy:** Proton pump inhibitors (PPIs; e.g. omeprazole class), NSAIDs and common analgesics, anti-inflammatories, aminosalicylates (5-ASA, mesalazine, sulfasalazine), and corticosteroids (systemic and topical, such as budesonide) as the mainstay of small-molecule IBD and GI co-medication literature [24,30].

**Systemic immunomodulatory therapy:** Anti-TNF agents (e.g. infliximab, adalimumab), integrin antagonists (vedolizumab, etrolizumab, natalizumab), IL-12/23 blockade (ustekinumab, risankizumab), and JAK inhibitors (tofacitinib, upadacitinib, filgotinib), together with classical immunosuppressants (thiopurines, methotrexate, calcineurin inhibitors, mycophenolate), define modern systemic immunomodulation in IBD [25,31].

**Supplementary Figures**

**Supplementary Figure S1.** Literature analysis workflow. (a) Filtering, text preprocessing, and embedding generation; (b) Logical expression (LOGEXP) framework; (c) Low coverage concept identification.

**Supplementary Figure S2**. Global dendrogram built from pairwise semantic distances in embedding space using the Neighbour-Joining (NJ) method, including n=1,500 leaves. The Global tree is meant to be read as a “map” of the main topics in the corpus; for each word, we check the corresponding articles linked to that word in the embedding space (assessing the output WORD.html). Note that the macro-groups were not created automatically: they were manually assigned by reviewing the abstracts to identify the topics they consistently discussed, and then grouping words that shared recurring ideas and terminology. A brief explanation of each macro-group is provided in the Supplementary Material. For the complete tree, see the **Data Availability** section. Collapsed subtrees were omitted for readability, as many branches represent broader IBD-related terminology outside the protease-serpin-microbiome axis.

**Supplementary Figure S3**. Distribution of nDocs values, with vertical dashed lines showing the 10th (P10), 15th (P15), and 20th (P20) percentiles. P15 was selected to identify low-coverage concepts, corresponding to concepts retrieved in up to 20 documents (nDocs ≤ 20). Concepts below this threshold tend to have sparser retrieval, less stable document embeddings due to limited evidence, and higher sensitivity to query formulation. In contrast, concepts above this range exhibit more consistent coverage and more stable retrieval behaviour.

**Supplementary Figure S4.** UPGMA dendrogram of the LOGEXP concepts shows seven groups of related concepts, including comparatively under-explored themes (stars indicate concepts with low coverage, nDocs ≤ 20). Leaf labels are colored according to thematic dimensions (i-v).

`

**Supplementary Figure S5.** Pairwise semantic distance between LOGEXP concepts in embedding space. Lower-triangular heatmap of the symmetric 49 × 49 Euclidean distance matrix between LOGEXP mean vectors. Each LOGEXP embedding was computed as the average of document-level SWeeP embeddings across all nDocs retrieved for that concept. Darker cells indicate smaller distances (greater similarity between associated literatures), while lighter cells indicate larger separation. Rows and columns follow the leaf ordering of the UPGMA dendrogram (**Supplementary Figure S4**). Dimensions (i-v) are shown in the column adjacent to the heatmap.

# **Supplementary References**

1. Iliev ID, Ananthakrishnan AN, Guo CJ. Microbiota in inflammatory bowel disease: mechanisms of disease and therapeutic opportunities. Nat Rev Microbiol. 2025;23:509–524. doi:10.1038/s41579-025-01163-0

2. Deraison C, Vergnolle N. Proteases in intestinal health and disease. Nat Rev Gastroenterol Hepatol. 2026;23:6–28. doi:10.1038/s41575-025-01129-w

3. Lloyd-Price J, Arze C, Ananthakrishnan AN, et al. Multi-omics of the gut microbial ecosystem in inflammatory bowel diseases. Nature. 2019;569:655–662. doi:10.1038/s41586-019-1237-9

4. Rooks MG, Garrett WS. Gut microbiota, metabolites and host immunity. Nat Rev Immunol. 2016;16:341–352. doi:10.1038/nri.2016.42

5. Roager HM, Licht TR. Microbial tryptophan catabolites in health and disease. Nat Commun. 2018;9:3294. doi:10.1038/s41467-018-05470-4

6. Michaudel C, Danne C, et al. Rewiring the altered tryptophan metabolism as a novel therapeutic strategy in inflammatory bowel diseases. Gut. 2023;72(7):1296–1310. doi:10.1136/gutjnl-2022-327337

7. Ridlon JM, Kang DJ, Hylemon PB, Bajaj JS. Bile salts and the gut microbiome. Curr Opin Gastroenterol. 2014;30:332–338. doi:10.1097/MOG.0000000000000056

8. Wahlström A, Sayin SI, Marschall HU, Bäckhed F. Intestinal crosstalk between bile acids and microbiota and its impact on host metabolism. Cell Metab. 2016;24:41–50. doi:10.1016/j.cmet.2016.05.041

9. Buisson A, Denizot J. Adherent-invasive Escherichia coli (AIEC) in Crohn’s disease: where are we in 2025? Clin Res Hepatol Gastroenterol. 2025;49:102727. doi:10.1016/j.clinre.2025.102727

10. Barnich N, Darfeuille-Michaud A. Role of bacteria in the etiopathogenesis of inflammatory bowel disease. World J Gastroenterol. 2007;13:5571–5576. doi:10.3748/wjg.v13.i42.5571

11. O’Sullivan S, Gilmer JF, Medina C, Hudson SP. Matrix metalloproteinases in inflammatory bowel disease: an update. Mediators Inflamm. 2015;2015:964131. doi:10.1155/2015/964131

12. Biel C, et al. Matrix metalloproteinases in intestinal fibrosis. J Crohns Colitis. 2023;17:ijad178. doi:10.1093/ecco-jcc/ijad178

13. Kriaa A, Jablaoui A, Mkaouar H, et al. Serine proteases at the cutting edge of IBD: focus on gastrointestinal inflammation. FASEB J. 2020;34:7270–7282. doi:10.1096/fj.202000031RR

14. Wang Y, Han J, Yang G, et al. Therapeutic potential of the secreted Kazal-type serine protease inhibitor SPINK4 in colitis. Nat Commun. 2024;15:5874. doi:10.1038/s41467-024-50048-y

15. Mkaouar H, Mariaule V, Rhimi S, et al. Gut serpinome: emerging evidence in IBD. Int J Mol Sci. 2021;22(11):6088. doi:10.3390/ijms22116088

16. Su L, et al. TNFR2 activates MLCK-dependent tight junction dysregulation and immune activation in the intestinal epithelium. Nat Med. 2013;19:1643–1650. doi:10.1038/nm.3359

17. Cenac N, et al. Role for protease activity in visceral pain in irritable bowel syndrome. J Clin Invest. 2007;117:636–647. doi:10.1172/JCI29255

18. Turner JR. Intestinal mucosal barrier function in health and disease. Nat Rev Immunol. 2009;9:799–809. doi:10.1038/nri2653

19. Mosli MH, Zou G, Garg SK, et al. C-reactive protein, fecal calprotectin, and stool lactoferrin for detection of endoscopic activity in symptomatic inflammatory bowel disease patients: a systematic review and meta-analysis. Am J Gastroenterol. 2015;110:802–819. doi:10.1038/ajg.2015.120

20. Norman JM, Handley SA, Baldridge MT, et al. Disease-specific alterations in the enteric virome in inflammatory bowel disease. Cell Host Microbe. 2015;18:307–320. doi:10.1016/j.chom.2015.09.008

21. Richard ML, Lamas B, Liguoro D, et al. Mycoses in inflammatory bowel diseases: analyses of in situ colonization of the gastrointestinal tract in a prospective cohort. J Crohns Colitis. 2018;12:619–628. doi:10.1093/ecco-jcc/jjy020

22. Paramsothy S, Kamm MA, Kaakoush NO, et al. Multidonor intensive faecal microbiota transplantation for active ulcerative colitis: a randomised placebo-controlled trial. Lancet. 2017;389:1218–1228. doi:10.1016/S0140-6736(17)30182-4

23. Turner D, Ricciuto A, Lewis A, et al. STRIDE-II: an update on the Selecting Therapeutic Targets in Inflammatory Bowel Disease (STRIDE) Initiative of the International Organization for the Study of IBD (IOBD). J Crohns Colitis. 2021;15:1351–1360. doi:10.1093/ecco-jcc/jjab109

24. Baumgart DC, Sandborn WJ. Inflammatory bowel disease: clinical aspects and established and evolving therapies. Lancet. 2007;369:1641–1657. doi:10.1016/S0140-6736(07)60727-X

25. Torres J, Bonovas S, Doherty G, et al. ECCO Guidelines on Therapeutics in Crohn’s disease: medical treatment. J Crohns Colitis. 2020;14:4–44. doi:10.1093/ecco-jcc/jjz187

26. Shaw SA, Blanchard JF, Bernstein CN. Association between the use of antibiotics in the first year of life and pediatric inflammatory bowel disease. Am J Gastroenterol. 2010;105:2683–2690. doi:10.1038/ajg.2010.260

27. Lupp C, Robertson ML, Wickham ME, et al. Host-mediated inflammation disrupts the intestinal microbiota and promotes the overgrowth of Enterobacteriaceae. Cell Host Microbe. 2007;2:119–130. doi:10.1016/j.chom.2007.08.002

28. Nguyen LH, Örtqvist AK, Cao Y, et al. Antibiotic use and risk of inflammatory bowel disease: a nationwide, population-based study in Sweden. Clin Gastroenterol Hepatol. 2020;18:1577–1585. doi:10.1016/j.cgh.2019.10.028

29. Atarashi K, Tanoue T, Shima T, et al. Induction of colonic regulatory T cells by indigenous Clostridium species. Science. 2011;331:337–341. doi:10.1126/science.1198469

30. Harbord M, Eliakim R, Bettenworth D, et al. Third European evidence-based consensus on diagnosis and management of ulcerative colitis. Part 2: Current management. J Crohns Colitis. 2017;11:769–784. doi:10.1093/ecco-jcc/jjx009

31. Danese S, Furfaro F, Peyrin-Biroulet L. Review article: the clinical pharmacology of anti-TNF agents in inflammatory bowel disease. Aliment Pharmacol Ther. 2019;49:380–391. doi:10.1111/apt.15112

32. Albenberg LG, Wu GD. Diet and the intestinal microbiome: associations, functions, and implications for health and disease. Gastroenterology. 2014;146:1564–1572. doi:10.1053/j.gastro.2014.01.058

33. Hooper LV, Littman DR, Macpherson AJ. Interactions between the microbiota and the immune system. Science. 2012;336:1268–1273. doi:10.1126/science.1223490

34. Schirmer M, Denson LA, Vlamakis H, et al. Compositional and temporal variation in the mucosally adherent human fecal microbiota. Cell Rep. 2018;24:832–844. doi:10.1016/j.celrep.2018.06.084

35. Pascal V, et al. A microbial signature for Crohn’s disease. Gut. 2017;66:813–822. doi:10.1136/gutjnl-2016-313235

36. Akiyama S, et al. Multi-biome analysis identifies distinct gut microbial signatures and their crosstalk in ulcerative colitis and Crohn’s disease. Nat Commun. 2024;15:10291. doi:10.1038/s41467-024-54797-8

37. Jiang SS, Chen YX, Fang JY. Fusobacterium nucleatum: ecology, pathogenesis and clinical implications. Nat Rev Microbiol. 2026;24:197–214. doi:10.1038/s41579-025-01237-z

38. Clayton EM, et al. The vexed relationship between Clostridium difficile and inflammatory bowel disease: an assessment of carriage in an outpatient setting among patients in remission. Am J Gastroenterol. 2009;104:1162–1169. doi:10.1038/ajg.2009.4

39. Alotaibi AD, et al. Prevalence of H. Pylori in inflammatory bowel disease patients and its association with severity. BMC Gastroenterol. 2025;25:317. doi:10.1186/s12876-025-03892-1
